# Relationship between gamer profiles, gaming behavior, sociodemographic characteristics, and big five personality traits among French law students

**DOI:** 10.1186/s40359-023-01329-6

**Published:** 2023-09-22

**Authors:** Germano Vera Cruz, Anne-Marie Barrault-Méthy, Marion Del Bove, Michael Nauge

**Affiliations:** 1 Department of Psychology, UR7273 CRP-CPO, University of Picardie Jules Verne, Campus Chemin du Thil, Amiens, 80000 France; 2 Department of Law, U. Bordeaux, Bordeaux, 4600 CERFAPS France; 3 UR15076 FoReLLIS, University of Poitiers, Poitiers, France; 4 Linguistics Research Center - Corpus, Discourse and Societies, University Jean Moulin Lyon 3, Lyon, France

**Keywords:** Players typologies, Gaming behavior, Law students, BFI personality traits

## Abstract

**Background:**

Over the past 10 years, gamer profiles have been developed to understand the reason underlying players’ intrinsic motivation. While the research undertaken has led to the creation of distinct models (e.g., BrainHex and Hexad typologies), there is a lack of studies on the prevalence of these profiles among a specific population and the association between the target population’s profiles and their personality traits, gaming behavior, and sociodemographic characteristics.

**Methods:**

Thus, the present study aimed to (a) establish the gamer profiles of French undergraduate law students, (b) examine the relationships between the participants’ profiles and their personality traits, gaming behavior, and sociodemographic characteristics, with a view to the development of serious games specifically intended for this population. In total, 753 French undergraduate students participated in the study, completing an online questionnaire. Data were analyzed using Latent Profile Analysis (LPA), chi-square, and ANOVA.

**Results:**

The main findings show that among participants, the two most prevalent gamers’ profiles are Mastermind (45%) and Seeker (22.7%); followed by three less represented archetypes: Conqueror (12.9%), Daredevil (9.7%), and Achiever (9.7%). These archetypes are associated with the participants’ Big Five personality traits. Specifically, Daredevils, Masterminds, and Seekers have high mean scores on Extraversion (*p* < .001); Achievers and Seekers have high mean scores on Agreeableness (*p* < .001); Seekers and Achievers have high mean scores on Neuroticism (*p* < .001); and Seekers, Masterminds, and Achievers have high mean scores on Openness (*p* < .001). The unveiled profiles are also significantly associated with the participants’ gaming behavior including their playing frequency (p < .001), game types (*p* = .031), and sociodemographic characteristics (*p* < .001). For example, Masterminds are more likely to be female than the other four profiles (*p* < .001), while Conquerors and Daredevils are more likely to have a low socio-economic status compared to those with intermediate and high socio-economic status (*p* = .49).

**Conclusion:**

These findings can be used to design serious/educational games tailored to the studied population.

**Supplementary Information:**

The online version contains supplementary material available at 10.1186/s40359-023-01329-6.

## Introduction

Typologies and personality traits seek both to understand how individuals differ from one another in cognition, emotion, motivation, and behavior, and explain the causes of such differences.

While the development of personality typologies and traits has been underway since the end of the 19th century [1], the development of gamer typologies models mainly began during the 1990s. In fact, Bartle’s [2] pioneer studies made it possible to understand that there is an extensive range of different personalities associated to the gamers’ intrinsic motivations, reasons to play, satisfaction with the game, etc. Such understanding has led to the development of different player typologies and to the development of games (for entertainment, competition, for educational or clinical purposes, etc.) associated with different potential player profiles.

Historically, Bartle [2] created one of the first players categorization. After analyzing the attitudes of the players of a game called Multi-User Dungeon (MUD])^1^, Bartle [2] theorized four player typologies: (1) *Achievers,* motivated by progressing and reaching a high level of proficiency, they seek to master a certain technical gesture or strategy and look for challenges and rewards which help them to move forward; (2) *Killers*, who are motivated by the idea of competing against others, they prioritize a good ranking or a victory above all else; (3) *Explorers*, who are motivated by exploration and discovery, they are comfortable with games that offer vast worlds and universes to discover and enjoy constantly discovering new games; (4) *Socializers,* who are motivated by the desire to share experiences, they enjoy playing with others, cooperating and collaborating with other people.

Since Bartle’s typology was developed from experiments on a specific game (MUD), game researchers in subsequent years began developing new typologies based on patterns of play, archetypes from neurobiological research, a body of literature on game emotions, previous typology approaches, and players’ intrinsic motivation [3–5]. Below, we present the two most cited models of players typologies [3, 4] developed by researchers in the past 15 years. For an overview on all the players typologies developed by researchers after Bartle’s study [2], see the meta-analysis by Sezgin [5].

In 2015, Andrzej Marczewski created a model of player types based on the intrinsic motivation called Hexad gaming typology [3, p. 65–80]: (1) *Socializers*, who are motivated by relatedness and want to interact with others and create social connections; (2) *Free spirits*, who are motivated by autonomy and self-expression and want to create and explore; (3) *Achievers*, who are motivated by mastery and are looking to learn new things and improve themselves, seeking challenges to overcome; (4) *Philanthropists*, who are motivated by purpose and meaning, and are altruistic, wanting to give to others and enrich their lives without expecting anything in return; (5) *Players*, who are motivated by rewards and will do what is necessary to collect rewards from a system, mainly interested in their own gains; (6) *Disruptors*, who are motivated by change and want to disrupt systems, either directly or through other users, to force positive or negative change. The questionnaire used to assess Haxed gamer typology was validated by Tondello et al. [6].

Following a game personality survey launched in 2009, a group of researchers created the BrainHex test, which comprises seven gamer typologies associated with some neurophysiological mechanisms that explain an individual gamer’s profile reinforcement and maintenance [4]: (1) *Seekers*, they like to experiment, enjoy open-world games, like finding alternative routes, and pride themselves on being the first to discover features; (2) *Survivors*, they enjoy experiencing moments of terror that trigger a state of excitement and arousal; (3) *Daredevils*, they enjoy the thrill of the chase, the excitement of taking risks, and generally like playing on the edge; (4) *Masterminds*, motivated by a problem that requires complex decision-making and strategy to overcome obstacles; for instance, they enjoy solving puzzles and concocting strategies; (5) *Conquerors*, challenge-oriented, they dislike winning easily, like overcoming adversity, and act forcefully, “channeling their anger in order to achieve victory” [4, p. 2] and the reward that comes with it; (6) *Socializers*, they enjoy spending time with other gamers and sharing the experiences of being in communion with them, cooperating, and talking game strategies; (7) *Achievers*, they are explicitly goal-oriented and motivated by long-term achievement. To classify individuals into BrainHex types and obtain their main class and subclass, the authors developed a questionnaire that participants can complete on a web platform to receive their classification automatically. The questionnaire used to assess an individual BrainHex dominant and secondary architypes was validated by Busch et al. [7].

### Players’ typologies, personality traits, and gaming behavior

In a study based on BrainHex model, Mailok et al. [8] found that the most dominant characteristics of digital games played by children aged 8–10 years old are Achiever (in games in which users strive to pursue the highest score), Daredevil (in games that are highly challenging), and Conqueror (in games that demand empowerments and struggles). Particularly, male children preferer to play games with the characteristics of Achiever (80.26%), Daredevil (80.26%), Conqueror (77.63%), Socializer (64.47%), Mastermind (57.89%), Survivor (48.68%), and Seeker (40.79%) compared to female children who tend to prefer playing games with the characteristics of Achiever (85.51%), Daredevil (60.14%), Mastermind (58.70), Seeker (55.07%), Conqueror (52.90%), Survivor (39.84%), and Socializer (28.98%) [8]. Using the BrainHex players’ typology, Birk at al. [9] found that players-centric traits (competence, autonomy, relatedness, presence, and intuitive control) are associated to Mastermind and Achievers; Zeigler-Hill and Monica [10] showed that extraversion personality trait (captured by the HEXOCO model [11]) was association with Daredevil and Socializer gaming preference. Using the Big Five model of personality [12], a study by Braun et al. [13] suggests that participants who preferred action games had high extraversion and low neuroticism. Regarding the Hexad gamer typology, findings from recent studies revealed that the most common types are Philanthropists, Achievers, and Free Spirits, followed by Socializers and Players, while the least common user type was Disruptors; women tended to score higher than men on the Disruptor user type [14]. In addition, from the Hexad model, Tondello at al. [15] concluded that Philanthropist was positively correlated with extraversion, agreeableness, conscientiousness, and openness; Socializer type was positively correlated with extraversion and with agreeableness; Free Spirit was positively correlated with openness and with extraversion, but negatively with neuroticism; Achiever was positively correlated with conscientiousness; Disruptor was negatively correlated with neuroticism; Player was positively correlated with conscientiousness.

### Players’ typologies and de development of modern games

The development of different models of player typologies, particularly the three mentioned above, has directly or indirectly influenced the development of modern games on three axes [5, 16, 17]. The first axis involves the development of entertainment games tailored to specific consumers, based on playful activities that generate a perceived challenge sufficient for players to enjoy and engage with [17, 18]. The goal is to create games that cater for different groups of players’ demographics, enabling a personalized experience and thus increasing sales and profits [17, 19, 20]. The second axis is the development of educational games that facilitate learning processes and activities. Learners often abandon learning environments that are not tailored to their particular cognitive, motivational, and emotional patterns [5, 17, 21]. Gamification is currently being developed as a game-based learning approach to enhance learners’ motivation effectively. The third axis is the development of therapeutic/clinical games that induce cognitive and behavioral restructuring/capacity and attenuate the manifestation of certain disorders or symptoms [5, 17, 20].

To our knowledge, there is currently limited literature available on the prevalence of gamers’ typologies in different population groups, which could inform the development of game-based learning programs or therapeutic/clinical interventions [5]. For example, there is a lack of scientific information on player profiles among students based on their field of study, as well as a gap in understanding the relationship between socio-demographic characteristics, gaming behavior, player typologies; and personality traits.

### The present study

#### Purpose

The present study aims to (a) establish the gamer profiles of French undergraduate law students and (b) examine the relationships between the participants’ gamer profiles and their gaming behavior, sociodemographic characteristics, and personality traits.

It must be noted that in this study, “players typologies” or “archetypes” and “game profiles” are used interchangeably. Specifically, “player typologies” are used to refer to the archetypes included in the theoretical model upon which the current study is based, while “game profiles” are used as a general reference to the classification of the participants into the modeled player typologies.

#### Research questions

The study’s purpose was divided into four research questions:

1. What gamer typologies (profiles = class and subclass) are prevalent among french undergraduate law students?.
2. What are the relationships between the participants’ gamer profiles and their gaming behavior?.
3. What are the relationships between the participants’ gamer profiles and their sociodemographic characteristics?.
4. What are the effects of the participants big five personality traits on their gamer profiles?.

As this was conceived as exploratory study, we did not elaborate any hypotheses associated to the four research questions.

Finally, we chose to base our study on the BrainHex model of player’ typology [4] rather than the Marczewski model [3]. We made this choice because the former is mainly founded on archetypes from neurobiological research, while the latter is based on players’ intrinsic motivation. We assumed that a typology model based on neurobiological research would be more likely to report stable individual characteristics [4, 5]. Therefore, it would be more pertinent to examine the relationships between these “stable” game profiles and the Big-five personality traits, which are also considered to be neurobiologically grounded and stable over time [12, 22–24]. Moreover, this choice was made based on the assumption that an archetype model theoretically grounded in neurobiological research would constitute a more pertinent theoretical justification for using it as the basis for the secondary purpose of the current study: the creation of a pedagogic serious game for French undergraduate law students. Additionally, as the BrainHex model [4] preceded the Marczewski typology [3], we decided to conduct a study based on the former typology first and consider a similar study based on the latter typology in the near future.

## Methods

### Participants

In total, 753 undergraduate law students from two French universities (University of Bordeaux and University of Lyon) participated in the study.

The participants’ age ranged from 17 to 26 years (*M* = 19.93, *SD* = 1.58). The participants’ sex distribution was as follows: female = 533, male = 220. Tables 1 and 2 display all the participants’ gaming behavior and sociodemographic characteristics.

**Table 1:** Relationship Between Participants Gamer Typologies and their Gaming Behavior

Game preferences	Gamer typologies					Total (n/%)
	Mastermind *n* = 339	Seeker *n* = 171	Conqueror *n* = 97	Daredevil *n* = 73	Achiever *n* = 73	753 (100%)
**Player types** (X^2^ = 45.55, df = 8, p < .001, CV = 0.24)						
Game averse	36 (10.6%)^a^	12 (7.0%)^a^	8 (8.2%)^a^	7 (9.6%)^a^	21 (28.8%)^a^	84 (11%)
Occasional player	204 (61.0%)^a^	79 (46.2%)^a^	60 (61.9%)^a,b^	36 (49.3%)^a^	37 (50.7%)^a^	416 (55%)
Die-hard player	99 (29.4%)^a^	80 (46.8%)^a,b^	29 (29.9%)^a^	30(41.1%)^a^	15 (20.5%)^a^	253 (34%)
**Gaming frequency** (X2 = 35.79, df = 8, p <. 001, CV = 0.21)						
Rare	88 (26.0%)^a,^	34 (19.9%)^a^	25 (25.8%)^a,^	11 (15.1%)^a^	27 (37.0%)^a^	185 (25%)
Occasional	155 (45.7%)^b^	57 (33.3%)^a^	47 (48.5%)^a^	32 (43.8%)^a^	34 (46.6%)^a^	325 (43%)
Frequent	96 (28.3%)^c^	80 (46.8%)^a^	25 (25.8%)^a^	30 (41.1%)^a^	12 (16.4%)^a^	243 (32%)
**Game testes** **(**X^2^ = 16.96, df = 8, p = .031, CV = 0.15)						
Digital games	38 (12.1%)^a^	36 (21.8%)^a^	15 (16.7%)^a,^	14 (20.3%)^a^	9 (15.3%)^a^	112 (16%)
Real life games	98 (31.2%)^a^	30 (18.2%)^a^	19 (21.1%)^b^	18 (26.1%)^a^	19 (32.2%)^a^	184 (26%)
Both	178 (56.7%)^a^	99 (60.0%)^a^	56 (62.2%)^c^	37 (53.6%)^a^	31 (52.5%)^a^	401 (58%)
**Game device used** (X2 = 6.80, df = 8, p = .558, CV = 0.070)						
Smartphone or tablet	3 (1.0%)^a^	3 (1.8%)^a^	2 (2.2%)^a^	0 (0,0%)^a^	0 (0.0%)^a^	8 (1%)
Computer or console	70 (22.3%)^b^	34 (20.6%)^b^	14 (15.6%)^a^	12 (17,4%)^a^	16 (27.1%)^a^	146 (21%)
Both	241 (76.8%)^c^	128 (77.6%)^c^	74 (82,2%)^a^	57 (82.6%)a	43 (72.9%)^a^	543 (78%)
**Social play behavior** X^2^ = 19.22, df = 12, p = .083, CV = 0.09						
Alone	14 (4.4%)^a^	6 (3.5%)^a^	12 (12.5%)^a^	6 (8.2%)^a^	4(5.6%)^a^	42 (6%)
In cooperation with others	52 (16.2%)^b^	22 (12.9%)^a^	17 (17.7%)^a^	18 (24.7%)^a^	13 (18.3%)^b^	122 (17%)
In competition with others	63 (19.6%)^c^	32 (18.8%)^a^	15 (15.6%)^a^	14 (19,2%)^a^	15 (21.1%)^c^	139 (19%)
No preferences	192 (59.8%)^d^	110 (64.7%)^a^	52(54.2%)a	35 (47.9%)^a^	39 (54.9%)^d^	428 (58%)
**Game scenario** X^2^ = 40.16, df = 8, p < .001, CV = 17						
Prefers without scenario	57 (17.7%)^a^	14 (8.2%)^a^	10 (10.3%)^a^	12 (16.4%)^a^	23 (31.9%)^a^	116 (16%)
Indifferent	10 (3.1%)^b^	12 (7.0%)^a^	0 (7.0%)^a^	0 (0.0%)^b^	0 (0.0%)^a^	22 (3%)
Prefers with scenario	255(79.2%)^c^	145(84.8%)^a^	87(89.7%)^a^	61(83.6%)^c^	49(68.1%)^a^	597(81)

CV = Cramer’s V

Data are shown as *n* (%). In the column direction, figures with the same exponent in each column are significantly different (*p* < .05). For example: regarding Game scenario, 84.8%% is significantly different from 7.0% and from 8.2%; 83.6%, 0.0%, and 16.4% are not significantly different; they have different exponents. In the row direction, variables relationships are given by the X^2^ in parentheses

**Table 2:** Associations Between Socio-demographic Characteristics and Participants’ Dominant Gaming Typologies

Characteristics	Gamer typologies					Total (n and %)
	Masterm *n* = 339	Seeker *n* = 171	Conqueror *n* = 97	Daredevil *n* = 73	Achiever *n* = 73	753 (100%)
Sex (X^2^ = 29.89, df = 4, p < .001, CV = 0.21)						
male	61 (19.8%)^a^	74 (37.9%)^a^	33 (30.0%)^a^	30 (36.1%)^a^	22 (27.5%)^a^	220 (29.2%)
female	247 (80.2%)^a^	105 (62.1%)^b^	70 (70.0%)^b^	53 (63.9%)^b^	58 (72.5%)^b^	533 (70.8%)
Age (X^2^ = 2.56, df = 4, p = .634, CV =. 060)						
17–21 years-old	254 (86.1%)^a^	151 (88.3%)^a^	84 (87.5%)^a^	61 (83.6%)^a^	65 (91.5%)^a^	615 (87%)
22–26 years-old	41 (13.9%)^b^	20 (11.7%)^b^	12 (12.5%)^b^	12 (16.4%)^b^	6 (8.5%)^b^	91 (13%)
SES (X^2^ = 15.14, df = 8 p = .049, CV = 0.11)						
low	23 (7.9%)^a^	11 (6.5%)^a^	11 (11.8%)^a^	12 (16.7%)^a^	6 (8.7%)^a^	63 (10%)
intermediate	222 (76.3%)^b^	127 (75.1%)^b^	75 (80.6%)^a^	55 (76.4%)^a^	52 (75.4%)^b^	531 (76%)
high	46 (15,8%)^c^	31 (18.3%)^c^	7 (7.5%)^a^	5 (6.9%)^a^	11 (15.9%)^c^	100 (14%)
Believers (X^2^ = 6.42, df = 8, p = .600, CV = 0.068						
No	140 (47.6%)^a^	78 (45.9%)^a^	44 (45.8%)^a^	32 (44.4%)^a^	36 (51.4%)^a^	330 (47%)
Yes	107 (36.4%)^b^	63 (37.1%)^b^	30 (31.3%)^a^	30 (41.7%)^b^	27 (38.6%)^a^	257 (37%)
Agnostic	47 (16.0%)^c^	29 (17.1%)^c^	22 (22.9%)^a^	10 (13.9%)^c^	7 (6.1%)^b^	115 (16%)
Practicing believers (X^2^ = 2.93, df = 4, p = .569, CV = 0.086)						
Non	96 (62.7%)^a^	62 (68.1%)^a^	35 (68.6%)^a^	23 (57.5%)^a^	19 (55.9%)^a^	235 (64%)
Yes	57 (37.3%)^b^	29 (31.9%)^b^	16 (31.4%)^b^	17 (42.5%)^b^	15 (44.1%)^b^	134 (36%)

Masterm = Mastermind; CV = Cramer’s V; SES = Socioeconomic status

Data are shown as *n* (%) and above the expected values. In column direction, figures with the same exponent in each column are significantly different (*p* < .05). For example: regarding the Believer variable, 22.9% is significantly different from 31.3% and 45.8%, they have the same exponent; 16.0%, 36.4%, and 47.6% are not significantly different; they have different exponents. In the row direction, variables relationships are given by the X^2^ in parentheses

### Recruitment and sampling

Participants were recruited in their classrooms by their university professors. The inclusion criteria were (a) “anyone who is undergraduate law students” in the designated universities and (b) “who is willing to participate in the study”. No particular sampling or participant selection technics were used. The minimum number of participants required (n = 335) was fixed at 5 by the total number of items (67) in the two scales used for data collection (as recommended by Wolf at al. [25]).

### Data collection material

The data collection materials used in this study are presented in the supplementary material 2 associated with this article. The data collection material comprise four questionnaires.

#### Gamer profiles questionnaire (GPQ)

The gamer profile questionnaire used in this study was developed from the translation and adaptation of the BrainHex questionnaire [4]. It consists of 21 items, which corresponds to seven dimensions or typologies: *Mastermind*, *Seekers*, *Daredevil*, *Conqueror*, *Achiever*, *Socializer*, and *Survivor*. Each dimension has 3 items. Each of the 21 items was preceded by the instruction “Please rate each videogame experience listed. Choose from a scale between ‘I hate it!’ (for experiences you would rather avoid) to ‘I love it!’ (for experiences you would be happy to go through)”, and followed by a five-point response scale: 1 (I hate it!) to 5 (I love it!). Two item samples are: “Playing in a group, online or in the same room”, “Be at the wheel of a vehicle going at full speed”. Additionally, the questionnaire included a 22nd item that asked participants to rank seven statements, indicating an equal number of gaming moments which were designed to refer to the seven gamer typologies modeled in the study. Examples of the statements that participants had to rank include: “A moment when you feel an intense sense of unity with another player” and “A moment of breathtaking speed or vertigo”.

It is important to note that the 21 items were designed to categorize participants into one of the seven players typologies based on their responses (scores on each item scale). The 22nd item was designed to directly identify the typologies with which participants most identified with, by ranking their preferred gaming moments.

In the present study, this questionnaire was validated though confirmatory factorial analysis (CFA) which had the following goodness of fit indices: x^2^/df = 4.53; CFI = 0.97; TLI = 0.96; SRMR = 0.052; RMSEA = 0.034. The internal reliability (Cronbach’s alpha coefficient) by dimension was as follows: Mastermind = 0.72; Seeker = 0.68, Daredevil = 0.67, Conqueror = 71, Achiever = 74, Socializer = 77, and Survivor = 80. These metrics indicated good/acceptable psychometric properties [26, 27].

#### Gaming behavior questionnaire (GBQ)

The questionnaire used in this study consisted of six questions designed to measure the participants’ (a) gaming frequency, (b) gaming attitudes, (c) game type preference (digital, in real life [IRL], or both), (d) devices used to play games, (e) preference for playing alone, in cooperation, or in competition with other players, and (f) whether the game scenario mattered for their enjoyment of the game.

#### Sociodemographic characteristics questionnaire (SDCQ)

The questionnaire used in this study includes questions asking participants to indicate their (a) age, (b) gender (female, male, or other), socioeconomic status (SES: low, intermediate, or high)^2^, religious beliefs (no, yes, agnostic), and whether they were practicing believers (no, yes).

#### Big five inventory (BFI)

The participants’ personality traits were evaluated using the French version of the BFI [22]. This self-administered questionnaire consisted of 45 items that measured five dimensions: *Extraversion* (outgoing/energetic vs. solitary/reserved), *Agreeableness* (friendly/compassionate vs. challenging/callous), *Conscientiousness* (efficient/organized vs. extravagant/careless), *Neuroticism* (sensitive/nervous vs. resilient/confident) and *Openness to experience* (inventive/curious vs. consistent/cautious). Participants rated each item on a Likert scale ranging from 1 (strongly disagree) to 5 (strongly agree). Here are two item samples: “I see myself as someone who is helpful and not selfish with others”; “I see myself as someone who tends to be quiet”.

In the present study, this questionnaire was validated though CFA which had the following goodness of fit indices: x^2^/df = 5.67; CFI = 0.97; TLI = 0.98; SRMR = 0.036; RMSEA = 0.032. The internal reliability (Cronbach’s alpha coefficient) by dimension was as follows: Extraversion = 0.91, Agreeableness = 0.94, Conscientiousness = 76, Neuroticism = 88; Openness = 75. These metrics indicated good psychometric properties [26, 27].

To prevent participants from completing a set of four questionnaires at once, which would lead to a possible fatigue effect, data collection was organized in two stages. First, the participants received a link to complete the questionnaires GPQ, GBQ, and SDCQ. Two weeks later, the students who had completed the first set of online questionnaires received a code and the second link to a website for completed the BFI. It should be noted that, while 753 students complete the GPQ, GBQ, and the SDCQ, only 377 (females = 223, males = 154) of these 753 students completed the BFI. As result, some statistical analyses included 753 participants and other analyses included the 372 participants who completed all the questionnaires (five participants were eliminated for technical reasons, see data analysis sub-section for details).

### Procedure

The professors from two French university faculties of law (University of Bordeaux and University Jean Moulin Lyon 3) recruited participants in their classrooms. All participants were undergraduate students. The professors explained the purpose of the study and informed the students that they would receive two links to a website via email to complete a set of online questionnaires. Students were informed that participation was voluntary. Participants did not receive any remuneration for answering the questionnaires. The research was conducted according to the ethical committee of the University of Bordeaux requirements for this kind of studies.

### Potential recruitment bias

While the link to complete the first set of online questionnaires was send to all undergraduate students registered in the aforementioned universities at the time of the participants recruitment, the participation in the study was voluntary and anonym. No specific selection criteria or sampling procedures were applied. All target students could participate if they wanted. Also, it is important to note that the students’ professors who publicized the study were unable to know among their students who completed the questionnaires and who did not. Only 12.59% of the study target population completed the first set of online questionnaires. The raison why some target students decide to participate and others did not is unknow. It is possible that some target students, for technical raison, did not receive the link sent to them. Furthermore, between the first and the follow-up set of questionnaires to be completed, the attrition rate stood at 50.06%. The exact reasons for the relatively high level of attrition are also unknown; it may be related to a lack of time or lack of interest in continuing their participation. Finally, among the 753 participants, 70.78% identified as female; among the 377 participants, 59.15% identified as female. While we were enabled to access the official statistics on the number of the target population by sex, this may be explained by the fact that, in France, women represent 55.6% of higher education students and they account for 86.7% students on human, social and paramedical sciences [28].

### Data analysis

Before carrying out statistical analysis to respond to the research questions, we conducted normality checks on the variables of interest (the seven BrainHex dimensions and the five BFI dimensions). In addition, we conducted bivariate correlation analysis between the referred dimensions and the other study modeled variables.

#### Gamer profiles

To answer the first research question, we conducted a Latent Profile Analysis (LPA) on the data collected with the 21 items of the GPQ, using the R package *tidyLPA*. The data with all the 753 participants who completed the first set of the online questionnaires was used for this analysis. LPA is a person-oriented data analysis that attempts to identify groups/classes of individuals (latent profiles) based on responses to a set of continuous variables. LAP modeling assumes that there are “unobserved latent profiles that generate patterns of responses on indicator items” [29, p. 146]. LPA is a probabilistic model and a branch of Gaussian Finite Mixture Modeling, which means that it models the probability for an individual to belong to a given profile. The LPA approach differs from others, like K-mean clustering that uses distance algorithms (e.g., Euclidian distance). LPA is recommended in studies aiming to profile individuals based on behavioral and psychological measurements [30].

To estimate the ideal number of gamer profiles that best fit the data, we conducted nine LPA models. According to the Bayesian Information Criterion (BIC), the Entropy, and the Likelihood Ratio Test Statistic (LRTS) metrics, the model with five profiles (group classes) best fits the data. Because no grouping statistical model is perfect, the final profile of each participant was the result of an adjustment of their profile yielded by the LPA model and the ranking of the preferred gaming moments made by the participants (the 22nd item of the GPQ). More precisely, the LPA model enabled us to establish, for each participant, their two dominant archetypes (the class and the subclass), i.e., *Mastermind-Achiever*, based on the means and probabilities of belonging to these typologies. By examining the participants’ ranking of their preferred gaming moments (as a reminder, a ranking made according the 22nd item of the GPQ instructions and designed to indicate the participant’s preferred typologies, among the seven), we selected the three highest-ranked typologies (i.e., *Survivor-Mastermind-Achiever*). If the class and subclass from the LPA model were both present in the first three typologies as ranked by the participants, the profile from the LPA model was confirmed and upheld in the same class-subclass order. If only one of the typologies from the LPA model was confirmed and upheld in the top three ranked typologies as ranked by the participants, that typology was upheld as the class; then, the first typology from the participant’s ranking became the subclass profile (i.e., *Daredevil-Conqueror* [from de LPA model classification] compared to *Socializer-Daredevil-Survivor* [from the participants own ranking preference] = final participant profile *Daredevil-Socializer*). If neither the class nor the subclass was present in the participants’ top three ranked typologies, the first typology from the participant’s ranking became the profile class, and the class from the LPA model became the subclass (i.e., *Achiever-Seeker* compared to *Mastermind-Conqueror-Socializer* = final participant profile *Mastermind-Achiever*).

#### Associations between profiles and gaming behavior

To answer the second research question, we conducted a chi-square of independence analysis using SPSS statistics software (version 28). The data with all the 753 participants who completed the first set of the online questionnaires was used for this analysis.

#### Associations between profiles and sociodemographic characteristics

To answer the third research question, we conducted a chi-square of independence analysis using SPSS statistics software (version 28). The data with all the 753 participants who completed the first set of the online questionnaires was used for this analysis.

#### The effect of the big five personality traits on the gamer profiles

To answer the fourth research question, we conducted five between-subject one-way ANOVA tests examining the relationship between participants’ BFI personality traits (in the following order: Extraversion, Agreeableness, Consciousness, Neuroticism, and Openness) and their profile classes (five classes), using the SPSS statistics software (version 28). Among the 377 participants who completed the follow-up online questionnaires only 372 were included in this analysis. Five participate where excluded because of technical raisons (it was not possible to match their BrainHex profiles resulting from the analysis of the first set of questionnaires and their responses on the follow-up questionnaire [the BFI]).

#### Ponderation and significance level

The internal consistency of the dimensions of the GPQ and the BFI was assessed by Cronbach’s alpha coefficient, which was > 0.70 in both cases. When necessary, the data was weighted for the chi-square and ANOVA analyses. The significance level was set at p < .05.

## Results

The normality checks and the correlation analysis are summarized in Appendix 1 (respectively in Table A, Table B, and Table C). The results show that the data is normally distributed (see Skewness and Kurtosis values in Appendix 1, Table A). Excepting age that is negatively correlated to Achiever gamer type, the socio-demographic characteristics are not significantly associated neither to the players’ profiles nor to the personality traits.

### The students’ gamer profiles

**Fig. 1:**
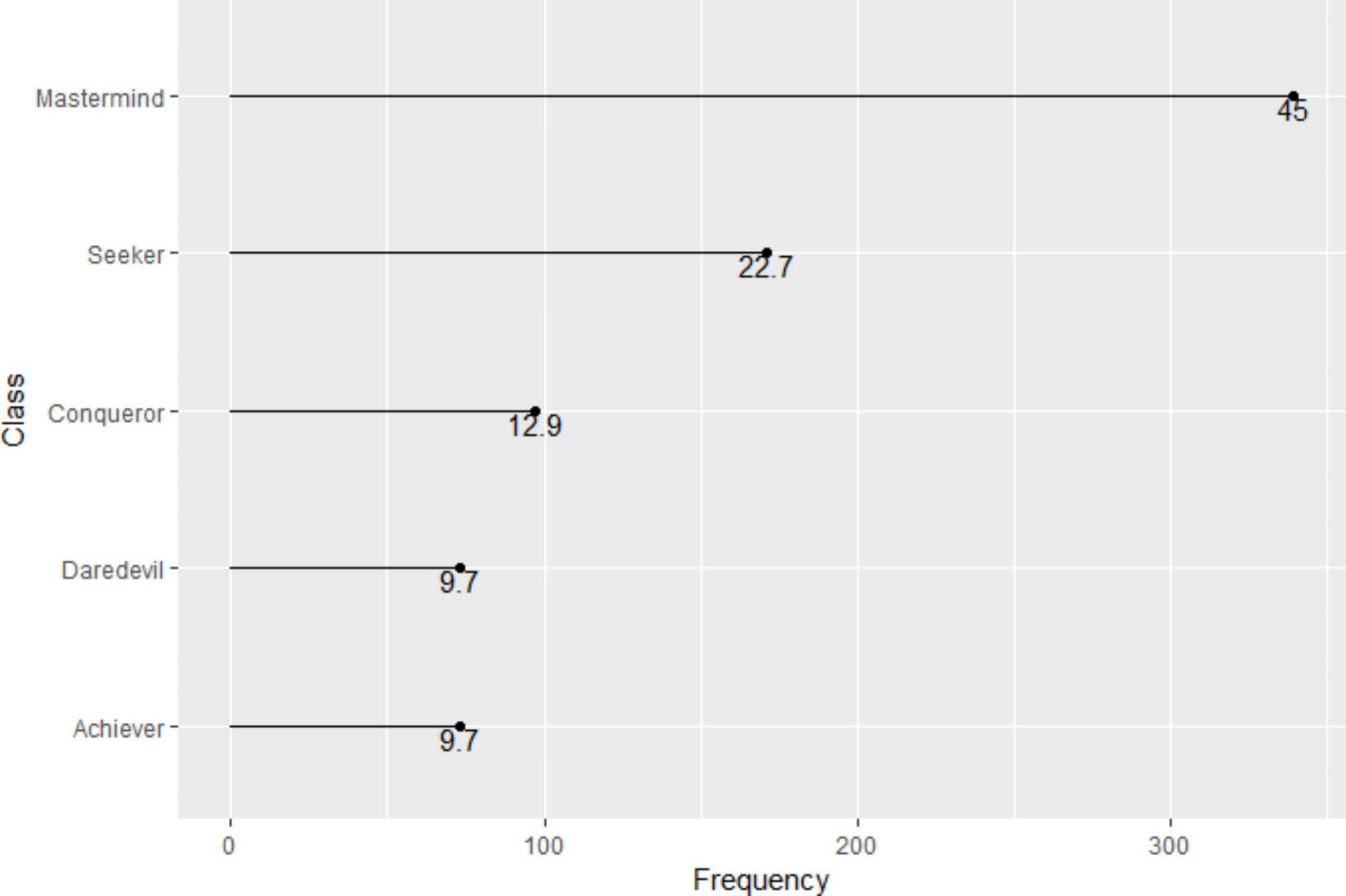
Participants’ main classes of belonging (game profiles). This figure presents the frequencies of individuals par gaming typology, out of 753 participants

**Fig. 2:**
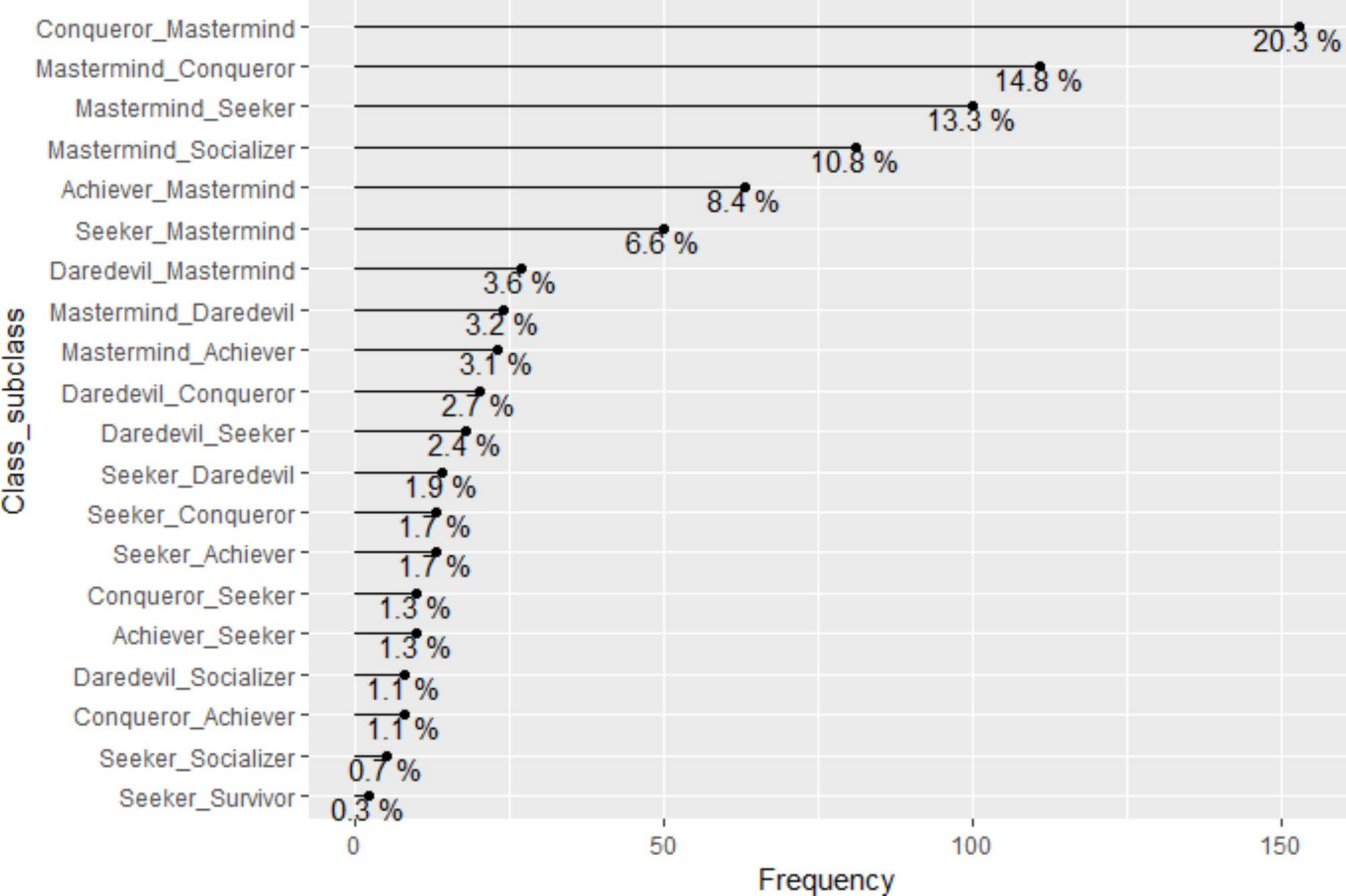
Participants’ class-subclass of belonging (game profiles). This figure displays the frequencies of individuals par gaming typology, out of 753 participants

As a result of the classification made, Fig. 1 shows the participants’ main gamer profiles (the class of belonging) and their respective frequencies. Figure 2 presents the participants’ double gamer profiles (the class-subclass of belonging) and their respective frequencies. As shown in Fig. 1, the study population was classified into five main profiles (classes: Mastermind = 339[45%], Seeker = 171[22.7%], Conqueror = 97[12.9%], Daredevil = 73[9.7%], Achiever = 73[9.7%]); and, as depicted in Figs. 2 and 20 sub-profiles (class-subclasses).

### Relationships between the participants’ profiles and their gaming behavior and sociodemographics

Table 1 presents the main results of the chi-squared analysis between the participants’ profiles and their reported gaming behavior.

All profiles were more likely to belong to the group of *occasional players* than to the *game-averse* or *die-hard player.*, Masterminds and Conquerors were significantly more likely to describe themselves as *occasional players* than the other profiles (for all comparisons, see Table 1 for the chi-squared statistics and significant relationships). Conquerors were more likely to *play occasionally* compared to other profiles, whereas Seekers and Daredevils were more likely to *play frequently*. Masterminds and Achievers prefer to play *real-life games* (RLG) over *digital games*, while Seekers and Daredevils preferred digital games to RLG. Achiever participants reported significantly more use of *computers and consoles* to play games than *smartphones or tablets* as game device, while there was no clear preference among the four other groups. Among Conquerors, significantly more individuals reported a preference for *playing alone* than *playing in cooperation* or *in competition* with other players. Surprisingly, among Daredevils, significantly more participants indicated a preference for playing in cooperation with other players than playing alone or in competition with other players. Finally, Achievers significantly preferred *playing games without scenarios* than to *playing games with scenarios*; conversely, Conquerors significantly preferred *playing games with scenarios* than to *playing games without scenarios*.

Table 2 presents the chi-squared analysis between participants’ gamer profiles and their sociodemographic characteristics.

The results of the chi-squared analysis revealed that Masterminds are significantly more likely to be *female* than the other four profiles. There was no significant difference in the likelihood of being male or female for Seekers, Conquerors, Daredevils, and Achievers. For more detailed comparisons, refer to the table for the chi-squared statistics and significant relationships.

Furthermore, the findings showed that there was no significant difference in the likelihood of belonging to the two age groups modeled (17–21 years-old vs. 22–26 years-old) for all profiles. However, Conquerors and Daredevils were significantly more likely to belong to the *low SES* than the *intermediate* or *high SES*. There was no significant association between the SES-modeled categories and the other three profiles.

In terms of religious beliefs, Achievers were significantly less likely to be *agnostic* compared to *believers* or *non-believers*. However, there was no significant relationship between religion and the other four participants profiles.

### The effects of personality traits on gamer profiles

Table 3 shows the ANOVA conducted between participants’ personality traits and their gamer profiles. The total variance explained by the five models was respectively R^2^ = 0.55, R^2^ = 0.53, R^2^ = 0.49, R^2^ = 0.56, R^2^ = 0.51. The Levene test of homogeneity statistics was respectively 2.47, *p* = .312; 1.68, *p* = .173; 1.45, *p* = .372; 4.14, *p* = .111; 2.44, *p* = .299.

**Table 3:** Main Results of ANOVAs Conducted Between the BFI Personality-traits and the five Participants’ Gamer Profile Classes

Personality-traits	df	MS	F	p	n^2^_p_
Extraversion	4	47.78	77.38	< 0.001	0.65
Agreeableness	4	18.48	46.12	< 0.001	0.53
Consciousness	4	2.70	5.81	0.062	0.28
Neuroticism	4	49.98	97.92	< 0.001	0.51
Openness	4	30.48	68.52	< 0.001	0.42

df = degree of freedom, MS = Mean Squared, F = variance ratio, p = probability, n^2^_p_ = effect-size

There was significant difference among the five participants’ gamer profiles on the Extraversion personality trait, *F*(4, 372) = 77.38, *p* < .001, n^2^_p_ = 0.56. Tukey HSD post-hoc testing revealed significant differences between Daredevils, Masterminds, Seekers (who had high Extraversion mean scores, respectively *M* = 4.23, *M* = 3.98, *M* = 3.80) and Conquerors and Achievers (who had relatively low Extraversion scores, respectively *M* = 2.26 and *M* = 2.45).

There were also significant differences among the five participants’ gamer profiles on the Agreeableness personality trait, *F*(4, 372) = 46.12, *p* < .001, n^2^_p_ = 0.45. Tukey HSD post-hoc testing revealed significant differences between Achievers, Seekers (who had high Agreeableness mean scores, respectively *M* = 3.77, *M* = 3.65) and Conquerors, Daredevils, and Masterminds (who had relatively low Agreeableness mean scores, respectively *M* = 3.12, *M* = 2.79, and *M* = 2.69).

However, there was no significant difference among the five participants’ gamer profiles on the Consciousness personality trait, *F*(4, 372) = 5.81, *p* < .062, n^2^_p_ = 0.052.

There were significant differences among the five participants’ gamer profiles on the Neuroticism personality trait, *F*(4, 372) = 97.92, *p* < .001, n^2^_p_ = 0.59. Tukey HSD post-hoc testing revealed significant differences between Seekers and Achievers (who had high Neuroticism mean scores, respectively *M* = 4.11, *M* = 4.07) and Conqueror, Mastermind, and Daredevil (who had relatively low Neuroticism mean scores, respectively *M* = 2.85 and *M* = 2.54, and *M* = 2.27).

Finally, there were significant differences among the five participants’ gamer profiles on the Openness personality trait, *F*(4, 372) = 68.52, *p* < .001, n^2^_p_ = 0.45. Tukey HSD post-hoc testing revealed significant differences between Seekers, Masterminds, Achievers (who had high Openness mean scores, respectively *M* = 3.76, *M* = 3.67, *M* = 3.59) and Conquerors and Daredevils (who had relatively low Openness mean scores, respectively *M* = 2.64 and *M* = 2.20).

## Discussion

The present study aimed to (a) establish the gamer profiles of French undergraduate law students, (b) examine the relationships between participants’ gamer profiles and their gaming behavior, sociodemographic characteristics, and personality traits.

### Game profiles prevalence: overrepresentation of masterminds and seekers

Strikingly, the findings showed the prevalence of Masterminds and Seekers among the study participants. Among the 753 participants, about two thirds (339) of them were classified as Masterminds, and 171 of them as Seekers. The remaining classes (Conquerors, Daredevils, and Achievers) were less represented. Furthermore, among the 20 class-subclass combinations identified, Masterminds and Seekers were present respectively in nine and eight of them, respectively as the main class or as a subclass. Socializers and Survivors were not among the main profiles (classes); they appeared only as a subclass, and even then, only in two and one combination class-subclass, respectively, and for a very small number of participants. Mastermind players are motivated by finding solutions to problems that require developing appropriate responses; they enjoy solving puzzles and devising strategies, as well as focusing on making the most efficient decisions [4, 7]. These tendencies may explain, at least in part, why there is a predominance of Masterminds among undergraduate law students who are studying to become lawyers, judges, legal advisers, etc. As part of their work, lawyers, judges, and legal advisers must find solutions to their clients’ problems, devise strategies to defend their clients, or, as judges, try to understand the strategies presented by lawyers or legal advisers [31, 32]. Regarding the overrepresentation of Seekers, one of the characteristics of this profile is that they like to make discoveries and to be the only ones to know certain things [4, 7]; here again, this tendency may well fit individuals who might be studying to become some sorts of “detectives” searching for “hidden truths”, unveiling unlawful behaviors and criminal activities [33]. This result contrasts with finding from previous study [8] conducted among children and based on the BrainHex players’ typology in which Achiever and Daredevil were the dominant architypes (however, Daredevil and Achiever were in fact the 4th and the 5th prevalent gamer types in the current study). Also, our findings defer from another study [14], based on the Hexad gamer taxonomy, showing that among participants Philanthropists and Achiever was the most prevalent profiles. The difference in finding suggests that the prevalence of a given profile(s) in a specific population varies according to the characteristics of the studied population.

### Gamer profiles and gaming behavior

According to the results of the current study, the associations between the participants’ main profiles and their gaming behavior are nuanced. In terms of the frequency of play, Masterminds and Conquerors reported playing less often than the other profiles, with Conquerors being more likely to *play occasionally* compared to other profiles. In contrast, Seekers and Daredevils were more likely to *play frequently*. Given that Seekers enjoy exploring new worlds and new games [4, 7], and that Daredevils enjoy the trill of taking chances [4, 7], it makes sense that they would play more frequently than the other profiles. Previous studies have shown that Seekers and Daredevils (BrainHex typology), Killer (Bartle’s typology), and Players (Hexad typology) were more likely to play frequently and more likely to develop gaming “addiction” [5, 10, 13]. To some extent, these studies seem to be in line with each other, since Players share similar characteristics with Seekers, and Killer share features with Daredevils (see introduction section).

Regarding game types, the surprising results is that in this study, Daredevils indicated a preference for playing in cooperation with other players rather than playing alone or in competition with other players. One would expect that Daredevils would prefer competition, because of the risks associated with losing and the probability of winning [4, 7]. Since we did not found research works corroborating or contradicting these particular findings, further studies are necessary to shed light on this result.

Regarding other modeled gaming behaviors, Achievers prefer *playing games without scenarios* than *playing games with scenarios*, while Conquerors preferred the opposite. It is possible that games with scenarios are generally more challenging to play, thus exerting an attraction on Conquerors, who are challenge-oriented. It is also possible that games without scenarios are goal achievement-oriented, which would attract Achievers, who are goal-oriented. Future studies should explore this specific association.

### Gamer profiles and sociodemographic characteristics

Overall, the association between the participants’ main profiles and their sociodemographic characteristics appears weak. However, the study results reveal some interesting relationships. Firstly, Mastermind individuals are more likely to be female than male. Historically, in France, the judicial profession has been dominated by males [34], and women who aspire to become lawyers, judges, or counselors may have to develop strategic tendencies and resolution in decision-making to overcome potential professional barriers associated with gender stereotypes and discrimination [34, 35]. This historical fact could explain why Masterminds are more likely to be women among law students. Further studies are required to better understand this association. A previous study based on Hexad model found that women are men likely than men to be Disrupters [14]. In one hand, Disrupters seem different from Mastermind in the since that they want to change their environment, while the latter want to master it much more than change it. In other hand, however, they might have common ground in the sense that both are keen to face obstacles to achieve their goals. In both cases, the fact that in the majority of current world societies, women are more likely to face social barriers compared to men [34, 35], may explain the need to be Mastermind and Disrupters among them.

Secondly, Conquerors and Daredevils are significantly more likely to belong to *low socioeconomic status (SES)* than *intermediate* or *high SES*. This finding may be partly linked to the fact that individuals with low SES might have to take risks (Daredevils) and put in more efforts (Conquerors) to achieve certain goals, compared to those from intermediate or high SES. Indeed, research has shown that individuals from low SES are generally more likely to engage in risky behavior compared to those from higher SES [36, 37].

### Personality traits and gamer profiles

Much more relevant are the effects of the participants’ Big Five personality traits on their main gamer profiles. For example, Daredevils, Masterminds, Seekers scored high on Extraversion; Achievers and Seekers scored high on Agreeableness; Seekers and Achievers scored high on Neuroticism; Seekers, Masterminds, Achievers scored high on Openness. These findings are partially corroborated by previous studies [10, 14] based in both BrainHex [10] and Hexad architypes [14].

Some personality researchers have suggested that sensitivity to sensorial information reward is the core function underlying Extraversion [23, 24, 38] and that sensitivity to incentive reward mediated by the dopaminergic system is the primary driver of Extraversion [23, 39]. The match between the Extraversion personality trait and the profiles of Seeker and Daredevil makes sense, as the former archetype is motivated by an interest mechanism, which relates to the brain area processing sensory information and memory association [24, 40]; and the latter archetype is mainly focused on thrill-seeking. The association between Extraversion and Masterminds might be linked to a common underlying brain mechanisms linked to decision-making strategies [4, 7, 23, 24], during which inherent reward may interact with the sensitivity to sensory information mediated by the dopaminergic system.

In this study, it appears that Achievers, who are motivated by long-term achievements, had high scores on the Agreeableness personality trait, which may be associated with their tendency for togetherness, cooperation, altruism, and empathy – these being the core functions underlying Agreeableness [4, 7, 23]. Seekers, who are motivated by the interest mechanism, had high scores on the Agreeableness personality traits, possibly for the same reasons as mentioned above.

Seekers, Masterminds, and Achievers had high Openness scores, an association that may be mediated by the fact that people high in openness are imaginative, curious, innovative, perceptive, thoughtful, and creative [12, 22–24]. These are characteristics maybe required to better explore the world around them, make informed decisions, or achieve long-term goals.

Individuals high in Neuroticism are prone to emotional responses to stress that triggers avoidant/defensive behavior, including panic, irritability, depression, anxiety, and so on. [23, 38]. This may explain why the present study found that Daredevils and Conquerors have low scores on the Neuroticism personality trait. More research is needed to explain why, according to the result of the current study, Seekers and Achievers participants have high Neuroticism scores.

## Limitation

The extent to which the sample is representative of the target population is unknown, which means that the generalizability of the results must be approached with caution. For instance, there is an overrepresentation of females in the sample, partly because female students were overrepresented in the target population [28], partly because female students were more willing to participate in the study compared to male students. We managed to overcome this imbalance by weighing the data, when necessary, before the statistical analysis. The criterion for forming the two age groups (17–22 vs. 22–26) was based on the brain development literature showing that the human brain reaches its maturity (capacity for judgment and management of pleasure-seeking impulses) after 21 years-old [41, 42]. It is possible that a different criterion for age group partition would have led to different specific findings.

## Conclusion

The main findings of the present study suggest that:

- Among French undergraduate law students, there is an overrepresentation of Mastermind and Seeker gamer profiles.
- These two profiles are mainly associated with Extraversion, Agreeableness, and Openness BFI personality traits.
- Some of the participants’ gaming practices, behaviors, and experiences measured in the present study appear to be associated with their gamer profiles. In contrast, very few significant associations were established between the participants’ sociodemographic characteristics and their gaming profiles.

To our knowledge, this is the first study presenting the prevalence of BrainHex-based players’ typologies not only among university students but also among any other population. Additionally, as far as we know, this is the first study to examine the relationships between BrainHex-related players typologies and participants’ Big-Five personality traits, gaming behaviors, and sociodemographic characteristics. Thus, the results of the study bring a valuable contribution to the studied subject.

The relevance of the findings can be stated as follows:

- These finding can be used to design educational games tailored to the current study’s target population. When designing such educational games, it is necessary to take into account the prevalence of game typologies unveiled in the present study, as well as the relationships between game profiles and gaming behavior.
- The results of the present study can be used as hypothesis for future studies. It is advisable that such future studies adopt mixed methods (combination of qualitative and quantitative methods) to examine the same relationships and try to build a prediction model that combines player typologies, personality traits, and gaming behavior.

### Electronic supplementary material

Below is the link to the electronic supplementary material.

Supplementary Material 1

Supplementary Material 2

## Data Availability

Material used in this study (in French) and the data supporting this finding are available from the corresponding author upon reasonable request.

## Abbreviations

LPA: Latent Profile Analysis
MUD: Multi-User Dungeon
GPQ: Gamer profiles questionnaire
GBQ: Gaming behavior questionnaire
IRL: In real life
SDCQ: Sociodemographic characteristics questionnaire
SES: Socioeconomic status
BFI: Big Five Inventory
BIC: Bayesian information criterion
LRTS: Likelihood ratio test statistics
ANOVA: Analysis of variance
IRLG: In real life games

## Footnotes

1 In which players try to find out as much as they can about a virtual world.

2 The participants were required to answer this question subjectively, that is, according to their own evaluation of their socio-economic situation.
