# Supplementary material for: Relationship between gamer profiles, gaming behavior, sociodemographic characteristics, and big five personality traits among French law students
Source: BMC Psychol. 2023 Sep 22;11:285. doi: 10.1186/s40359-023-01329-6 (PMC10515229; doi:10.1186/s40359-023-01329-6)
Supplement: Supplementary file 1 — Supplementary Material 1 [file 40359_2023_1329_MOESM1_ESM.docx]

**Appendix 1**

**Table A.** Descriptive statistics of two scales dimensions’ scores

| Dimensions | N | Scale | Min | Max | M | SD | Skewness | Kurtosis |
| --- | --- | --- | --- | --- | --- | --- | --- | --- |
| Players’ typology | | | | | | | | |
| Mastermind | 753 | 1-5 | 1 | 5 | 3.91 | .66 | -.42 | .23 |
| Seeker | 753 | 1-5 | 1 | 5 | 3.83 | .71 | -.44 | .27 |
| Daredevil | 753 | 1-5 | 1 | 5 | 3.54 | .80 | -.21 | -.14 |
| Conqueror | 753 | 1-5 | 1 | 5 | 3.44 | .89 | -.36 | -.27 |
| Achiever | 753 | 1-5 | 1 | 5 | 3.82 | .80 | -.70 | .32 |
| Socializer | 753 | 1-5 | 1 | 5 | 3.73 | .91 | -.62 | .03 |
| Survivor | 753 | 1-5 | 1 | 5 | 3.48 | .93 | -.39 | -.36 |
| Personality traits | | | | | | | | |
| Extraversion | 372 | 1-5 | 1.28 | 4.85 | 3.20 | .77 | .02 | -.50 |
| Agreeableness | 372 | 1-5 | 2.12 | 4.00 | 3.18 | .36 | -.52 | -.05 |
| Conscientiousness | 372 | 1-5 | 1.87 | 4.62 | 3.68 | .48 | -.52 | .44 |
| Neuroticism | 372 | 1-5 | 1.57 | 4.16 | 3.06 | .58 | -.34 | -.40 |
| Openness | 372 | 1-5 | 2.22 | 5.00 | 3.60 | .57 | -.11 | -.43 |

M = mean; SD = standard deviation.

When the skewness is between -0.5 and 0.5, the distribution is fairly symmetric. If the value is greater than +1, the distribution is right skewed. If the value is less than -1, the distribution is left skewed. If the Kurtosis value is greater than + 1, the distribution is leptokurtic; if the value is less than -1, the distribution is platykurtic.

**Table C.** Person zero-order correlation between the BrainHex typology dimensions and the sociodemographic characteristics

| Variables | 1 | 2 | 3 | 4 | 5 | 6 | 7 | 8 | 9 | 10 | 11 | 12 |
| --- | --- | --- | --- | --- | --- | --- | --- | --- | --- | --- | --- | --- |
| 1.Mastermind | 1 |  |  |  |  |  |  |  |  |  |  |  |
| 2.Seeker | .39^**^ | 1 |  |  |  |  |  |  |  |  |  |  |
| 3.Daredevil | .35^**^ | .44^**^ | 1 |  |  |  |  |  |  |  |  |  |
| 4.Conqueror | .41^**^ | .34^**^ | .52^**^ | 1 |  |  |  |  |  |  |  |  |
| 5.Achiever | .33^**^ | .48^**^ | .35^**^ | .44^**^ | 1 |  |  |  |  |  |  |  |
| 6.Socializer | .26^**^ | .32^**^ | .37^**^ | .45^**^ | .30^**^ | 1 |  |  |  |  |  |  |
| 7.Survivor | .34^**^ | .45^**^ | .53^**^ | .57^**^ | .31^**^ | .33^**^ | 1 |  |  |  |  |  |
| 8.Age | -.05 | -.05 | .02 | -.01 | -.08^*^ | .01 | -.01 | 1 |  |  |  |  |
| 9.Sex | -.01 | .01 | -.02 | -.00 | .01 | .03 | -.02 | -.01 | 1 |  |  |  |
| 10.Education level | -.01 | -.01 | -.08^*^ | -.04 | -.05 | -.04 | -.05 | .06 | -.01 | 1 |  |  |
| 11.Socio-economic status | .01 | -.04 | .01 | -.02 | -.04 | .01 | -.03 | -,01 | .00 | .05 | 1 |  |
| 12.Religious practitioner | .03 | .04 | .01 | .04 | .02 | -.02 | .04 | -.01 | .03 | -.01 | -.04 | 1 |

**Correlation is significant at the 0.01 level.

*Correlation is significant at the 0.05 level.

**Table C.** Person zero-order correlation between the BFI dimensions and the sociodemographic characteristics

| Variables | 1 | 2 | 3 | 4 | 5 | 6 | 7 | 8 | 9 | 10 |
| --- | --- | --- | --- | --- | --- | --- | --- | --- | --- | --- |
|  |  |  |  |  |  |  |  |  |  |  |
| 1.Agreableness | 1 |  |  |  |  |  |  |  |  |  |
| 2.Extraversion | .08 | 1 |  |  |  |  |  |  |  |  |
| 3.Consciousiness | .10 | .30^**^ | 1 |  |  |  |  |  |  |  |
| 4.Neuroticism | .01 | -.34^**^ | -.12 | 1 |  |  |  |  |  |  |
| 5.Openness | .01 | .31^**^ | .26^**^ | -.19^*^ | 1 |  |  |  |  |  |
| 6.Age | -.02 | .02 | .07 | .02 | -.01 | 1 |  |  |  |  |
| 7.Sex | .09 | .14 | .01 | .14 | .04 | .11 | 1 |  |  |  |
| 8.Education level | .04 | -.10 | .09 | .02 | -.04 | -.43^**^ | -.05 | 1 |  |  |
| 9.Socio-economic status | -.03 | .02 | .07 | .03 | .06 | -.09 | -.04 | .11 | 1 |  |
| 10.Religious practitioner | .05 | .01 | .13 | .13 | .11 | .14 | .11 | -.07 | -.04 | 1 |

**Correlation is significant at the 0.01 level.

*Correlation is significant at the 0.05 level.
