# Supplementary material for: Relationship between gamer profiles, gaming behavior, sociodemographic characteristics, and big five personality traits among French law students
Source: BMC Psychol. 2023 Sep 22;11:285. doi: 10.1186/s40359-023-01329-6 (PMC10515229; doi:10.1186/s40359-023-01329-6)
Supplement: Supplementary file 2 — Supplementary Material 2 [file 40359_2023_1329_MOESM2_ESM.pdf]

THE STUDY QUESTIONNAIRE

Note: This questionnaire was translated from French. Indeed, the study questionnaire was administrated in French, which is the official language in France, were the study toke place.

General questions on sociodemographic variables

Age__

Sex:
Male__
Female__
Other__

Indicate your university educational level__

Indicate your socio-economic status:
This is a subjective assessment that you must make based on the socio-economic means that you and/or your parents have to live.
Low__
Intermediary__
High__

Are you a believer (regardless of religion)?
Yes__
No__
I don't want to respond__

General questions about gaming patterns and preferences

Choose the statement that suits you best.
I consider myself to be:
An inveterate gamer__
Between an inveterate player and an occasional player / between an inveterate player and an occasional player__
An occasional player / an occasional player__
Reluctant to digital games__
Reluctant to games in general__

I'm playing:
Every day__
Every week__
Occasionally__

Rarely__
Never__

Multiple answers possible.
I like to play:
To digital games__
IRL (In Real Life) games (board games, board games, escape games, crossword puzzles, etc.)__

Rank in order of preference.
As for digital games, I play on:
Computer__
Cellphone__
Tablet__
Console__

I have access to a virtual reality headset (where you slip your mobile phone, or autonomous, such as Oculus Quest or another brand).
Yes__
No__
I don't know what a virtual reality headset is__

Many possible responses.
When it comes to digital games, I like to play:
Alone__
In cooperation__
Against other players__
Non-digital games, I like to play:
Alone
In cooperation__
In competition__
Many possible responses__

What I think of video game scenarios:
The scenario contributes in a very important way to my pleasure of playing__
The scenario can contribute to my enjoyment of playing__
The scenario is not important when I play__
I prefer games without a scenario__
I do not play video games, whether online, on console, tablet or mobile phone__

Name three games that represent what you love about games.
You can quote any game, not just games on phone, tablet, PC or console:

Gamer Typologies Questionnaire

To see the original items in English, download the article on:
https://www.academia.edu/24953974/Player_Type_Models_Towards_Empirical_Validation

Instructions

Veuillez évaluer chaque expérience de jeu vidéo répertoriée. Choisissez parmi une échelle entre "Je déteste ça !" (pour les expériences que vous préférez éviter) à "J'adore ça !" (pour les expériences, vous seriez heureux d'y aller).

1. Exploration à la recherche de quelque chose (escape game, chasse au trésor, jeu d'objets cachés, jeu de piste, etc.).
   Je déteste ça!   1   2   3   4   5   J'adore ça!

2. Essayer d'échapper à un ennemi effrayant.
   Je déteste ça!   1   2   3   4   5   J'adore ça!

3. Résoudre une énigme.
   Je déteste ça!   1   2   3   4   5   J'adore ça!

4. Combattre un adversaire redoutable.
   Je déteste ça!   1   2   3   4   5   J'adore ça!

5. Jouer en groupe, sur internet ou dans une même pièce.
   Je déteste ça!   1   2   3   4   5   J'adore ça!

6. Réagir rapidement à une situation stimulante.
   Je déteste ça!   1   2   3   4   5   J'adore ça!

7. Récupérer tout ce qui peut l'être dans une zone donnée.
   Je déteste ça!   1   2   3   4   5   J'adore ça!

8. Regarder autour de moi pour profiter du spectacle.
   Je déteste ça!   1   2   3   4   5   J'adore ça!

9. Être au volant d'un véhicule qui roule à pleine vitesse.

Je déteste ça!   1   2   3   4   5   J’adore ça!

10. Mettre en place une stratégie prometteuse.
    Je déteste ça!   1   2   3   4   5   J’adore ça!

11. Eprouver du soulagement après m'être échappé(e).
    Je déteste ça!   1   2   3   4   5   J’adore ça!

12. Choisir un adversaire difficile dans un combat contre un autre joueur / joueuse (humain).
    Je déteste ça!   1   2   3   4   5   J’adore ça!

13. Discuter avec d'autres joueurs, en ligne ou dans la même pièce.
    Je déteste ça!   1   2   3   4   5   J’adore ça!

14. Trouver ce dont j'ai besoin pour compléter une collection.
    Je déteste ça!   1   2   3   4   5   J’adore ça!

15. Me retrouver suspendu(e) au bord d'une falaise.
    I love it!   1   2   3   4   5   I hate it!

16. Me demander ce qui se cache derrière cette porte fermée.
    Je déteste ça!   1   2   3   4   5   J’adore ça!

17. Avoir peur, être effrayé(e) ou mal à l'aise.
    Je déteste ça!   1   2   3   4   5   J’adore ça!

18. Chercher seul(e) ce qu'il y a à faire.
    Je déteste ça!   1   2   3   4   5   J’adore ça!

19. Réussir une épreuve difficile après avoir raté à de nombreuses reprises.
    Je déteste ça!   1   2   3   4   5   J’adore ça!

20. Coopérer avec des étrangers.
    Je déteste ça!   1   2   3   4   5   J’adore ça!

21. Obtenir un 100% après avoir fait toutes les épreuves d'un jeu.
    Je déteste ça!   1   2   3   4   5   J’adore ça!

Classez par ordre de préférence les moments de jeu suivants (1 = celui que je préfère, 7 = celui que j’aime le moins)

Un instant de stupeur devant quelque chose de merveilleux ou de splendide__
Une expérience de terreur primitive__
Un instant de vitesse vertigineuse__
L'instant où vous trouvez brusquement la solution à une énigme difficile__
L'instant où vous gagnez après une bataille acharnée__
L'instant où vous éprouvez un sentiment de communion avec un autre joueur__
Un instant de complétude pour lequel vous avez fait des effort__

BFI-Questionnaire

Check the statement indicating how much you approve or disapprove of the statement.
I see myself as someone who...

	Disagree a lot				Agree a lot
1. Talks a lot	1	2	3	4	5
2. Notices other people’s weak points	1	2	3	4	5
3. Does things carefully and completely	1	2	3	4	5
4. Is sad, depressed	1	2	3	4	5
5. Is original, comes up with new ideas	1	2	3	4	5
6. Keeps their thoughts to themselves	1	2	3	4	5
7. Is helpful and not selfish with others	1	2	3	4	5
8. Can be kind of careless	1	2	3	4	5
9. Is relaxed, handles stress well	1	2	3	4	5
10. Is curious about lots of different things	1	2	3	4	5
11. Has a lot of energy	1	2	3	4	5
12. Starts arguments with others	1	2	3	4	5
13. Is a good, hard worker	1	2	3	4	5
14. Can be tense; not always easy going	1	2	3	4	5
15. Clever; thinks a lot	1	2	3	4	5

16. Makes things exciting	1	2	3	4	5
17. Forgives others easily	1	2	3	4	5
18. Isn’t very organized	1	2	3	4	5
19. Worries a lot	1	2	3	4	5
	Disagree a lot				Agree a lot
20. Has a good, active imagination	1	2	3	4	5
21. Tends to be quiet	1	2	3	4	5
22. Usually trusts people	1	2	3	4	5
23. Tends to be lazy	1	2	3	4	5
24. Doesn’t get upset easily; steady	1	2	3	4	5
25. Is creative and inventive	1	2	3	4	5
26. Has a good, strong personality	1	2	3	4	5
27. Can be cold and distant with others	1	2	3	4	5
28. Keeps working until things are done	1	2	3	4	5
29. Can be moody	1	2	3	4	5
30. Likes artistic and creative experiences	1	2	3	4	5
31. Is kind of shy	1	2	3	4	5
32. Kind and considerate to almost everyone	1	2	3	4	5
33. Does things quickly and carefully	1	2	3	4	5
34. Stays calm in difficult situations	1	2	3	4	5
35. Likes work that is the same every time	1	2	3	4	5

36. Is outgoing; likes to be with people	1	2	3	4	5
37. Is sometimes rude to others	1	2	3	4	5
38. Makes plans and sticks to them	1	2	3	4	5
	Disagree a lot				Agree a lot
39. Get nervous easily	1	2	3	4	5
40. Likes to think and play with ideas	1	2	3	4	5
41. Doesn’t like artistic things (plays, music)	1	2	3	4	5
42. Likes to cooperate; goes along with others	1	2	3	4	5
43. Has trouble paying attention	1	2	3	4	5
44. Knows a lot about art, music and books	1	2	3	4	5
